# Chorea-acanthocytosis masquerading as a progressive seizure disorder with apparent early immunotherapy responsiveness

**DOI:** 10.1136/bmjno-2025-001531

**Published:** 2026-03-24

**Authors:** Amarachi Nwamba, Beheshta Harghandiwal, Bryan Ceronie, Edward Needham, Elizabeth Hutchinson, Jonathan Cleaver, Adam Handel

**Affiliations:** 1 University of Oxford Medical Sciences Division, Oxford, UK; 2 University of Oxford Nuffield Department of Clinical Neurosciences, Oxford, UK; 3 Oxford University Hospitals NHS Foundation Trust, Oxford, UK; 4 University of Cambridge Department of Clinical Neurosciences, Cambridge, UK; 5 University of Oxford, Oxford, UK

**Keywords:** NEUROGENETICS, NEUROPHYSIOLOGY, MOTOR, APRAXIA, CLINICAL NEUROLOGY, NEUROIMMUNOLOGY

## Abstract

Chorea-acanthocytosis (ChAc) is a rare genetic disorder characterised by a hyperkinetic movement disorder, dystonia, cognitive and neuropsychiatric deficits and seizures.

We report the case of a 30-year-old patient who presented with a decade of episodic neurological dysfunction and seizures. The condition was initially suspected to be an immunotherapy-responsive seronegative autoimmune encephalitis but progressed to oromandibular dystonia, raising suspicion of a neurodegenerative condition. Neuroimaging showed bilateral caudate atrophy and acanthocytes were seen on blood film microscopy in association with raised creatine kinase. Genetic testing revealed our patient to be compound heterozygous for two pathogenic variants in the *VPS13A* gene and confirmed the diagnosis of ChAc.

This case highlights the importance of considering ChAc in the differential diagnosis of a progressive treatment-refractory seizure disorder in the context of oromandibular dystonia.

## Case report

 A 30-year-old female school teacher was referred to the Oxford Encephalitis Clinic. Her symptoms began as a 20-year-old with transient left upper limb paraesthesia following glandular fever, which resolved within a month. At the age of 25, she developed headaches and difficulty with lesson planning. Following this, she experienced an episode of confusion characterised by the performance of repetitive actions. The following day, she suffered a tonic-clonic seizure preceded by left upper limb sensory symptoms. Over the course of the week, symptoms escalated, with frequent generalised tonic-clonic seizures, often occurring in clusters of 3–5, and prominent neuropsychiatric symptoms including paranoia, agitation, hallucinations and heightened emotionality. The patient was subsequently admitted to hospital. Before this, she had been fit and well with no personal or family history of neurological disease or risk factors for epilepsy.

**Figure 1:**
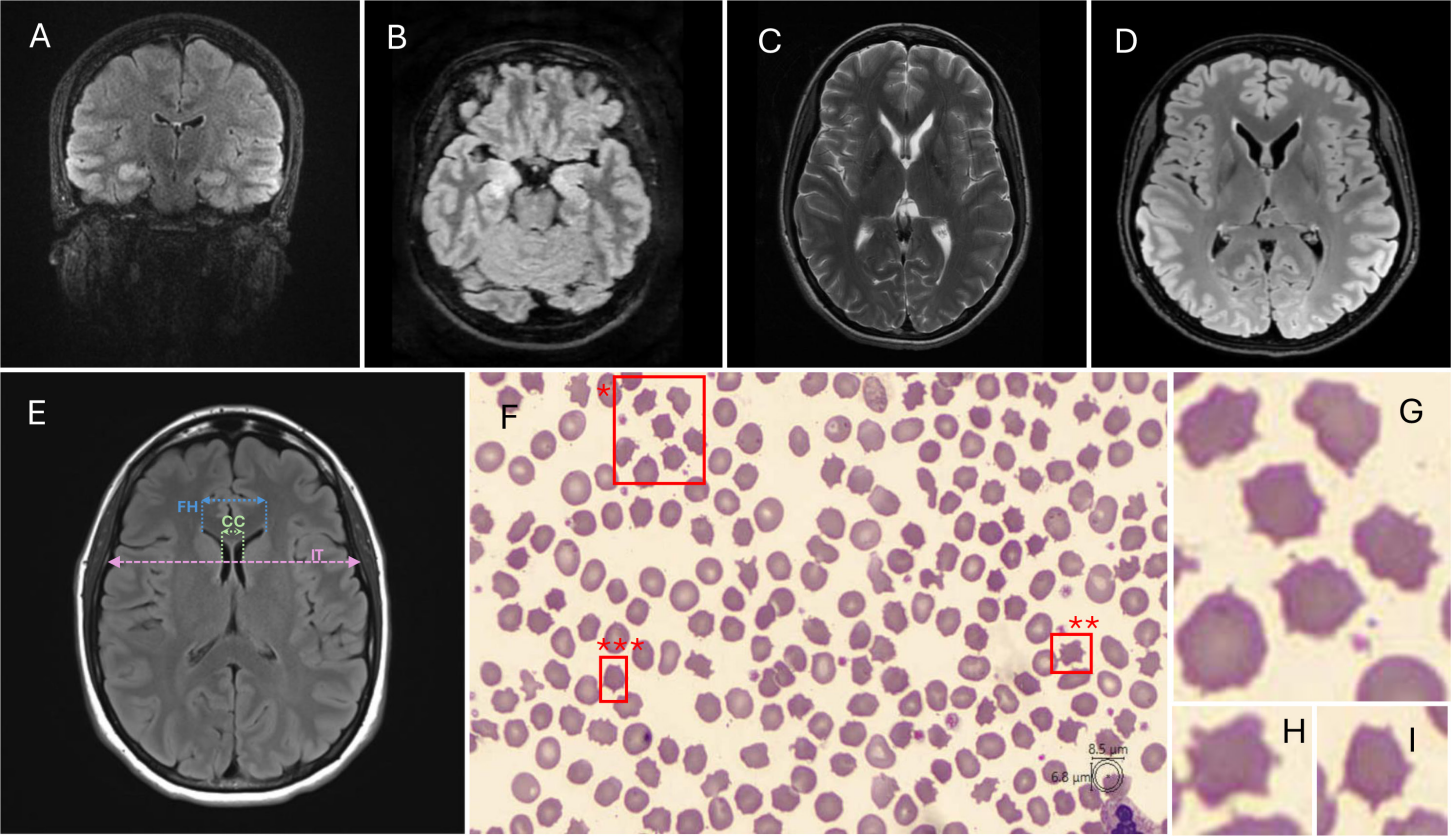
Paraclinical findings. An axial MRI brain illustrates high T2 signal within both hippocampi in 2019 (**A**) to 2023 (**B**). The patient’s imaging shows progressive caudate atrophy over time from 2020 (**C**) to 2024 (**D**). (**E**) Caudate atrophy can be objectively calculated using CC/IT or FH/CC ratios. (**F–I**) A blood film was performed showing irregularly spiculated projections (acanthocytes, boxed red) supporting the diagnosis of ChAc (**F**). *, ** and *** highlight acanthocytes, enlarged in (**G**), (**H**) and (**I**), respectively. CC, intercaudate distance; ChAc*, *chorea-acanthocytosis; FH, frontal horn width; IT, inner table width.

Cerebrospinal fluid (CSF) revealed normal white cells, protein and glucose. An MRI brain showed bilateral enlargement and T2 signal hyperintensity in the hippocampus and amygdala (figure 1), and an electroencephalogram demonstrated right temporal sharp waves. A broad autoantibody panel was negative for: Antinuclear antibody (ANA), N-methyl-D-aspartate receptor (NMDAR), Leucine-rich, glioma-inactivated 1 (LGI1), Contactin-associated protein-like 2 (CASPR2), γ-Aminobutyric acid sub-type A receptor(GABA_A_R), GABA_B_R, α-amino-3-hydroxy-5-methyl-4-isoxazolepropionic acid receptors (AMPAR), Dipeptidyl-peptidase-like protein 6 (DPPX), glycine, amphiphysin and paraneoplastic screen (most on more than one occasion). The patient was treated with intravenous methylprednisolone for presumed seronegative autoimmune encephalitis, after which her seizures stopped. However, her behaviour remained abnormal, so she underwent plasma exchange. The following additional investigations were also normal: immunoglobulins; lymphocyte subsets; ultrasound scan of ovaries and abdomen and CT scan of the thorax. Immunohistochemistry and live hippocampal rat neuron-based assays were performed on both serum and CSF, with negative results. Creatine kinase was elevated at 738 IU/L (range 29–168). Oral prednisolone treatment was continued following this admission and weaned over 6 months, over which time the patient made a good recovery.

The patient subsequently deteriorated 7 months later with executive dysfunction and personality changes including mood instability, concrete thinking and diminished empathic behaviour. 2 years on, memory deficits and further seizures emerged. This prompted re-treatment with prednisolone and the introduction of azathioprine while under the care of an outpatient neuroinflammatory clinic, with the patient showing clinical improvement. Aged 29, she presented again with seizures, which settled after treatment with methylprednisolone. MRI brain imaging at this stage revealed enlargement and T2/fluid-attenuated inversion Recovery (FLAIR) hyperintensity of hippocampi bilaterally, worse on the right and consistent with autoimmune encephalitis.

However, a month later, she was readmitted with apraxia of tongue movement which impaired both speech and swallowing. The emergence of this symptom, alongside the progressive nature of the condition, raised suspicion of a non-inflammatory aetiology. Around this time, she was unable to continue in her primary teaching role and transitioned to a teaching assistant position due to functional decline. The patient subsequently experienced further seizures along with memory difficulties and a reduction in ability to pick up social and non-verbal cues. Online supplemental figure demonstrates a timeline of symptom progression.

The patient was referred to the Oxford Encephalitis Clinic for suspicion of a relapsing neuroinflammatory condition. Neurological examination revealed prominent tongue apraxia, oromandibular dystonia, tongue motor impersistence and broken ocular saccades. Reflexes were present and symmetrical. The patient scored 87/100 on the Addenbrooke’s Cognitive Examination. CSF was acellular, with no neoplastic cells and was negative for NMDAR, AMPAR, LGI1 and CASPR2.

These findings, particularly tongue motor impersistence and oromandibular dystonia, raised clinical suspicion of neuroacanthocytosis; specifically chorea-acanthocytosis (ChAc). A blood film was requested to look for, and confirmed, presence of acanthocytes (figure 1). The patient’s brain imaging was reviewed in neuroradiology multidisciplinary team (MDT) meeting and retrospectively revealed caudate atrophy (figure 1).

Whole genome sequencing to detect single nucleotide variants, copy number variants and short tandem repeats was carried out for our patient, who was found to carry two heterozygous pathogenic vacuolar protein sorting-associated protein 13A (*VPS13A)* variants. Parental testing was not available; however, the clinical phenotype is highly consistent with ChAc.

**Variant 1**: NM_033305.2: c.2456C>G, p.Ser819*

This variant is predicted to result in a stop codon, leading to premature translation. It is predicted that this messenger RNA (mRNA) would be targeted for nonsense mediated decay. There is an established association between *VPS13A* loss of function variants and ChAc ^1^ (PVS1_Very Strong). This variant is very rare on the gnomAD population database v4.2 and^2^ (PM2_Moderate). This patient’s phenotype is highly specific for ChAc. *VPS13A* pathogenic variants are the only known cause (PP4_Supporting).^2^

**Variant 2**: NM_033305.2: c.6404dup, p.Ser2136Lysfs*2

This variant is predicted to result in a downstream stop codon, leading to premature termination of translation. It is predicted that this mRNA would be targeted for nonsense-mediated decay. (PVS1_Very Strong). This variant is very rare on the gnomAD population database v4.1 (PM2_Moderate).^2^ This variant has been reported before in the literature in an individual with a clinical diagnosis of ChAc and with an additional *VPS13A* pathogenic variant^3^ (PM3_Supporting). This patient’s phenotype is highly specific for ChAc. *VPS13A* pathogenic variants are the only known cause (PP4_Supporting).

Biallelic pathogenic *VPS13A* variants are associated with autosomal recessive movement disorders (predominantly orofacial choreic and dystonic movements), seizures, cognitive and behavioural changes and progressive distal muscle weakness.^4^

## Discussion

Neuroacanthocytosis encompasses a group of rare inherited disorders characterised by progressive neurological decline and the presence of abnormal, spiculated red blood cells known as acanthocytes. ChAc is one of the four neuroacanthocytosis syndromes, caused by mutations in the *VPS13A* gene on chromosome 9q21. It is exceedingly rare, with an estimated 500–1000 cases worldwide.^5^ The disease is marked by a hyperkinetic movement disorder, progressive cognitive decline, neuropsychiatric changes, dystonia and seizures.

This case illustrates the difficulty in recognising ChAc, especially early in the clinical trajectory. The seizure-predominant onset, along with apparent immunotherapy responsiveness, rendered the diagnosis particularly challenging. It also highlights the ability of ChAc to mimic other neurological conditions. The differentials in the appropriate clinical context include Huntington’s disease and Huntington-like disorders, seronegative autoimmune encephalitis, Wilson’s disease, conditions of neurodegeneration with iron accumulation and other neuroacanthocytosis syndromes.

In our case, the clinical constellation of seizures, neuropsychiatric symptoms and right hippocampal changes on MRI-steered clinical suspicion towards a seronegative autoimmune encephalitis. This was further supported by a positive response to immunotherapy, reinforcing a central nervous system autoimmune aetiology. In retrospect, steroid responsiveness may mask the progressive nature of the underlying neurodegenerative process. Additionally, corticosteroids may have exerted direct antiseizure effects^6^ potentially mediated through modulation of the Lyn kinase pathway.^7^ We highlight that steroid responsiveness alone does not necessarily imply an immune basis and should always be considered in the broader clinical context.

The inflection point in this patient’s diagnostic journey came with the emergence of an unusual apraxia of tongue movement deleteriously interfering with eating and speech. This oromandibular dystonia—specifically feeding dystonia, where the tongue expels food from the mouth on contact—is a harbinger of ChAc.^8^ In a cohort of 24 patients with neuroacanthocytosis, all demonstrated orofacial dystonia, with the majority exhibiting feeding dystonia, making it a pathognomonic sign of the condition.^9^ Its presence should prompt clinicians to consider neuroacanthocytosis, particularly in the context of seizures, cognitive change and movement abnormalities.^10^

Seizures are rarely seen as an isolated phenomenon in ChAc; however, they are a common symptom and may be the initial prominent feature, as in this case. In a cohort of nine patients, treatment-resistant seizures were the first prominent symptom of ChAc. Other symptoms, including dystonia and cognitive decline, would follow later and were often attributed to complications of epilepsy. This resulted in a significant diagnostic delay which exceeded a decade in some cases.^11^ Misdiagnoses of epilepsy therefore create a significant diagnostic delay.

While the presence of acanthocytes supports the diagnosis of ChAc, acanthocytosis is only observed in up to 50% of ChAc cases and can also present later in the clinical course.^12^ Moreover, acanthocytosis is not specific to but can be a marker of other neurological conditions including McLeod syndrome, Bassen-Kornzweig syndrome, familial hypobetalipoproteinaemia and others (online supplemental table). However, as a simple, minimally invasive test, it may be useful to consider blood films for patients with persisting neurological symptoms where the diagnosis is uncertain, as this may prompt genetic investigation. Ensuring a fresh blood sample used is also important to reduce false positives.

This case also denotes the critical role of re-reviewing imaging with a specific clinical hypothesis. The patient’s brain MRI was retrospectively reported to show caudate atrophy from index presentation. This is frequently observed in ChAc as a reflection of basal ganglia degeneration, a feature central to the disease. While not specific to ChAc by any means, these MRI findings are supportive when interpreted in the clinical context and may have pointed to a neurodegenerative aetiology earlier in the clinical course.

We recognise that this long diagnostic odyssey and coming to terms with a diagnosis of ChAc is a physically and emotionally challenging journey for our patient and many others. It is vital that an MDT approach is adopted to ensure that the holistic care of all the patient’s needs is provided.

## Supplementary material

10.1136/bmjno-2025-001531 online supplemental file 1

10.1136/bmjno-2025-001531 online supplemental file 2

## Data Availability

Data are available upon reasonable request.

## References

[1] Peikert K, Dobson-Stone C, Rampoldi L (2023). GeneReviews.

[2] Chen S, Francioli LC, Goodrich JK (2024). A genomic mutational constraint map using variation in 76,156 human genomes. Nature New Biol.

[3] Tomiyasu A, Nakamura M, Ichiba M (2011). Novel pathogenic mutations and copy number variations in the VPS13A gene in patients with chorea-acanthocytosis. Am J Med Genet B Neuropsychiatr Genet.

[4] Johns Hopkins University BMDWWWU (2026). Online mendelian inheritance in man, OMIM. https://omim.%20org.

[5] Jung HH, Danek A, Walker RH (2011). Neuroacanthocytosis syndromes. Orphanet J Rare Dis.

[6] Walsh R, Doherty CP, Doran E (2025). The use of steroids in adult epilepsy: A systematic review. Epilepsia Open.

[7] Peikert K, Federti E, Matte A (2021). Therapeutic targeting of Lyn kinase to treat chorea-acanthocytosis. Acta Neuropathol Commun.

[8] Bader B, Walker RH, Vogel M (2010). Tongue protrusion and feeding dystonia: a hallmark of chorea-acanthocytosis. Mov Disord.

[9] Danek A, Walker RH (2005). Neuroacanthocytosis. Curr Opin Neurol.

[10] Neeraja K, Prasad S, Holla VV (2021). The Spectrum of Movement Disorders in Neuroacanthocytosis Syndromes: A Video Series. Mov Disord Clin Pract.

[11] Benninger F, Afawi Z, Korczyn AD (2016). Seizures as presenting and prominent symptom in chorea-acanthocytosis with c.2343del VPS13A gene mutation. Epilepsia.

[12] Sorrentino G, De Renzo A, Miniello S (1999). Late appearance of acanthocytes during the course of chorea-acanthocytosis. J Neurol Sci.

