# Supplementary figures and images for: Chorea-acanthocytosis masquerading as a progressive seizure disorder with apparent early immunotherapy responsiveness
Source: BMJ Neurol Open. 2026 Mar 24;8(1):e001531. doi: 10.1136/bmjno-2025-001531 (PMC13034323; doi:10.1136/bmjno-2025-001531)

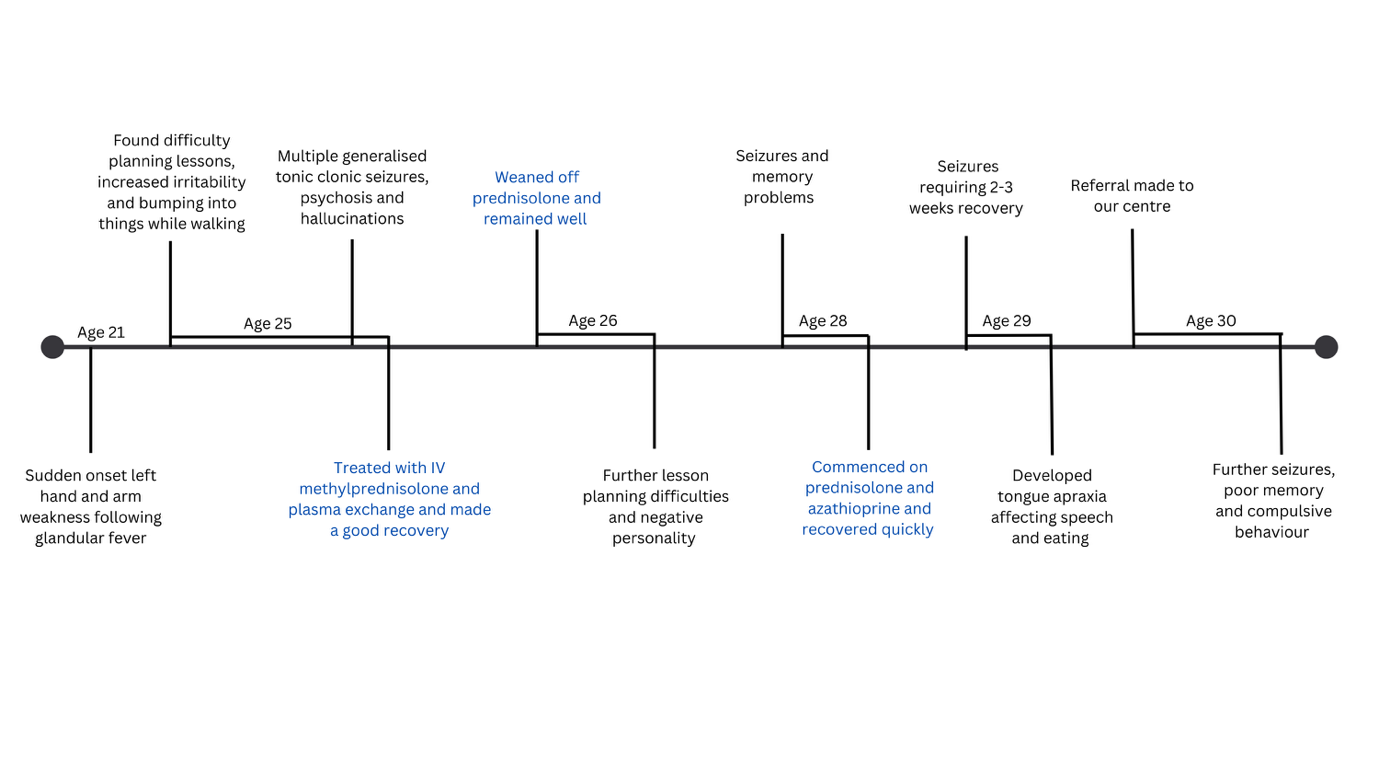

Supplement: online supplemental file 1 [file bmjno-8-1-s001.tiff]
