# Supplementary material for: Chorea-acanthocytosis masquerading as a progressive seizure disorder with apparent early immunotherapy responsiveness
Source: BMJ Neurol Open. 2026 Mar 24;8(1):e001531. doi: 10.1136/bmjno-2025-001531 (PMC13034323; doi:10.1136/bmjno-2025-001531)
Supplement: online supplemental file 2 [file bmjno-8-1-s002.docx]

**Supplemental** **Table**: Causes of oromandibular dystonia, motor impersistence, acanthocytosis and caudate atrophy.

| **Oromandibular dystonia** | **Acquired**   - Drugs and toxins   - Antidepressants (citalopram, sertraline, venlafaxine, fluoxetine, paroxetine)   - Antibiotics (cefixime, levofloxacin)   - Anaesthetic agents   - Methylphenidate   - Anticonvulsants - Metabolic/endocrine   - Kernicterus   - Extrapontine myelinolysis   - Uraemia   - Acquired hepatocellular encephalopathy - Autoimmune   - Behcet’s syndrome   - SLE   - Sjogren’s syndrome   - Autoimmune encephalitis (anti-NMDA, LGI-1, CASPR-2)   **Genetic**   - Autosomal dominant and X-linked   - *DYT-THAP1, DYT-GNAL*   - *DYT-TAF**   - *DYT-KMT2B**   - *DYT-ANO3** - Autosomal recessive   - Pantothenate kinase-associated neurodegeneration (PKAN)   - Wilson’s disease   - Chorea-acanthocytosis   - Aceruplasminaemia   - Niemann-Pick Disease Type C   Idiopathic isolated oromandibular dystonia |
| --- | --- |
| **Motor impersistence** | - Orolingual predominant   - Huntington’s Disease - Limb predominant   - Acute focal right-hemisphere stroke   - Callosal disconnection syndrome   - Diffuse cerebral or subcortical disorders |
| **Acanthocytosis** | - Inherited lipid disorders   - Abetalipoproteinemia *(*Bassen–Kornzweig)   - Familial hypobetalipoproteinaemia - Neuroacanthocytosis syndromes   - Chorea‑acanthocytosis (*VPS13A* mutation)   - McLeod syndrome (*XK* mutation)   - PKAN   - Huntington’s disease-like 2 - Acquired severe liver disease - Post‑splenectomy - Hypothyroidism - Myelodysplastic syndromes |
| **Caudate atrophy** | - Huntington’s Disease - Frontotemporal Lobar Degeneration - Alzheimer’s Disease - Temporal Lobe Epilepsy |
| *Oromandibular dystonia is a rare, though documented feature | |
